# From Mesenchymal Stromal/Stem Cells to Insulin-Producing Cells: Immunological Considerations

**DOI:** 10.3389/fimmu.2021.690623

**Published:** 2021-06-23

**Authors:** Ayman F. Refaie, Batoul L. Elbassiouny, Malgorzata Kloc, Omaima M. Sabek, Sherry M. Khater, Amani M. Ismail, Rania H. Mohamed, Mohamed A. Ghoneim

**Affiliations:** ^1^ Nephrology Department, Urology and Nephrology Center, Mansoura, Egypt; ^2^ Biotechnology Department, Urology and Nephrology Center, Mansoura, Egypt; ^3^ Department of Immunobiology, The Houston Methodist Research Institute, Houston, TX, United States; ^4^ Department of Surgery, The Houston Methodist Hospital, Houston, TX, United States; ^5^ Department of Genetics, The University of Texas, M.D. Anderson Cancer Center, Houston, TX, United States; ^6^ Department of Cell and Microbiology Biology, Weill Cornell Medical Biology, New York, NY, United States; ^7^ Pathology Department, Urology and Nephrology Center, Mansoura, Egypt; ^8^ Immunology Department, Urology and Nephrology Center, Mansoura, Egypt; ^9^ Biochemistry Department, Faculty of Science, Ain Shams University, Cairo, Egypt; ^10^ Urology Department, Urology and Nephrology Center, Mansoura, Egypt

**Keywords:** mesenchymal stem cells, insulin-producing cells, immunomodulation, immunogenicity, diabetes mellitus

## Abstract

Mesenchymal stem cell (MSC)-based therapy for type 1 diabetes mellitus (T1DM) has been the subject matter of many studies over the past few decades. The wide availability, negligible teratogenic risks and differentiation potential of MSCs promise a therapeutic alternative to traditional exogenous insulin injections or pancreatic transplantation. However, conflicting arguments have been reported regarding the immunological profile of MSCs. While some studies support their immune-privileged, immunomodulatory status and successful use in the treatment of several immune-mediated diseases, others maintain that allogeneic MSCs trigger immune responses, especially following differentiation or *in vivo* transplantation. In this review, the intricate mechanisms by which MSCs exert their immunomodulatory functions and the influencing variables are critically addressed. Furthermore, proposed avenues to enhance these effects, including cytokine pretreatment, coadministration of mTOR inhibitors, the use of Tregs and gene manipulation, are presented. As an alternative, the selection of high-benefit, low-risk donors based on HLA matching, PD-L_1_ expression and the absence of donor-specific antibodies (DSAs) are also discussed. Finally, the necessity for the transplantation of human MSC (hMSC)-derived insulin-producing cells (IPCs) into humanized mice is highlighted since this strategy may provide further insights into future clinical applications.

## Introduction

Type 1 diabetes mellitus (T1DM) accounts for approximately 5% of all diabetes cases and occurs as the result of the destruction of pancreatic islets through an autoimmune-mediated process. Patients typically depend on exogenous insulin injections throughout their lives. Glycaemic control can otherwise be achieved by β-cell replacement *via* the transplantation of the entire pancreas or its islets. Despite the increasing success of these methods, their application is limited by organ availability and the need for lifelong immunosuppression.

Recent progress in the field of regenerative medicine provides an alternative approach whereby surrogate β-cells can be generated from various stem cell sources. To this end, mesenchymal stromal/stem cells (MSCs) provide several distinct advantages, as they are widely obtainable from several tissues and have been reported to be safe and well tolerated with negligible teratogenic risk. MSCs can be selected on the basis of certain standards proposed by the Mesenchymal and Tissue Stem Cell Committee (1). Evidence was also provided that MSCs can be differentiated *in vitro* into insulin-producing cells (IPCs). This can be achieved by gene transfection with relevant endocrine genes (2), gene editing (3), or directed differentiation, whereby MSCs are cultured in a glucose-rich medium with a variety of activation and growth factors (4–7). In experimental animals, the efficiency of human MSC (hMSC)-derived IPCs in controlling chemically-induced diabetes was comparable to, if not better than, that of pluripotent stem cell-derived β-cells (8). An intriguing feature of MSCs is their ability to evade immune recognition. This immune-privileged status is attributed to their lack of expression of Human Leukocyte Antigen (HLA) class II, as well as the costimulatory molecules CD40, CD80 and CD86. Since MSCs can be harvested, expanded and stored for use, allogeneic MSCs are more practical for clinical applications than other cell types. However, many questions remain: will allogeneic hMSC-derived IPCs retain their immunomodulatory properties or become immunogenic after differentiation? And will they be susceptible to the detrimental effects of the *in vivo* microenvironment that destroyed native β-cells in type 1 diabetic patients after transplantation? This review will critically address these issues.

## Allogeneic MSCs Are Immunomodulatory

The immunomodulatory properties of MSCs were recognized approximately 2 decades ago. Bartholomew and associates reported one of the earliest *in vitro* and *in vivo* studies (9). When added to mitogen-stimulated lymphocytes, MSCs inhibited lymphocyte proliferation by approximately 50%. *In vivo*, the administration of MSCs to mismatched recipient baboons resulted in prolonged allograft survival of a third-party skin graft. These observations were further supported by several follow-up studies (10–14). Based on these findings, the immunomodulatory functions of hMSCs were exploited for the treatment of refractory immune-mediated diseases. Of these, the most frequently studied included graft-versus-host disease (15), aplastic anaemia (16), multiple sclerosis (17), rheumatoid arthritis (18), Crohn’s disease (19) and systemic lupus erythematosus (20).

## Allogenic MSCs Are Immunogenic

In contrast, a number of studies questioned the immunomodulatory capacity of allogenic MSCs and advocated that these cells are instead immunogenic (21, 22). Berglund and colleagues emphasized the importance of proper controls for matched or mismatched major histocompatibility complex (MHC) when investigating the immunogenicity of allogeneic MSCs (23). The researchers reported that MHC-mismatched MSCs evoked cell-mediated and humoral immune responses *in vivo*. In another study, Poncelet et al. showed that allogeneic porcine MSCs, which exhibited no immunogenicity *in vitro*, elicited an immune response following intracardiac injection (24). Furthermore, some reports argued that MSCs may acquire immunogenicity after differentiation. Lohan and associates demonstrated that, following MSC differentiation, the expression of immunological markers was induced and the secretion of immunomodulatory molecules, specifically prostaglandin E2 (PGE2), was suppressed (25). Similarly, Ryan et al. showed that allogeneic MSC-derived chondrocytes developed enhanced susceptibility to lysis by T cells following transplantation (26).

## The Special Case of Stem Cell-Derived IPCs

The immunogenicity of human umbilical cord-derived MSCs (hUC-MSCs) before and after directed differentiation to IPCs was investigated by Yang et al., both *in vitro* and *in vivo*. The authors reported that hUC-MSC-derived IPCs were hypoimmunogenic *in vitro* but evoked an allogeneic response after their transplantation in immune-competent mice (27). In a similar experiment, Hassanin et al. differentiated MSCs isolated from human umbilical cord Wharton’s jelly into IPCs (28). The researchers noted that the differentiated cells exhibited weak immunogenicity *in vitro*. However, following transplantation into chemically-induced diabetic rats *via* the portal vein, an immune response was induced. It is surprising to assume that the immunomodulatory capacity of MSCs could be effective across such a wide xenogeneic spectrum. In another study by van der Torren et al., human embryonic stem cell (hESC)-derived pancreatic progenitors were encapsulated and transplanted into NOD/SCID mice for further differentiation (29). Following maturation (20-25 weeks), the engrafted endocrine cells were explanted for *in vitro* immunogenicity testing. They observed that the differentiated cells became immunogenic compared to their hypoimmunogenic progenitors. We suggest that such an experiment would have been more informative if humanized mice had been utilized. On another note, a review by Sordi and colleagues reported that autologous induced pluripotent stem cells (iPSCs) and their differentiated IPCs were immunogenic (30). Such an observation was attributed to epigenetic modifications resulting from reprogramming and differentiation. However, Wood et al. highlighted several reports demonstrating reduced iPSC immunogenicity following differentiation (31). The authors also noted that the type of differentiated cells could influence the potential immunogenicity. According to Melton, a vigorous autoimmune response is expected following the transplantation of autologous iPSC-derived islets (32). Recently, in an *in vitro* investigation, Mohammadi and associates explored the immunogenicity of murine bone marrow-derived MSCs (BM-MSCs) after their differentiation to IPCs (33). They noted that differentiated MSCs acquired immunogenic properties. Such *in vitro* experiments, however, overlook the influence of the *in vivo* microenvironment.

Overall, the immunogenicity of pluripotent stem cell-derived IPCs has been confirmed. Their utilization in the clinical setting should, therefore, be within an immunoisolation device. On the other hand, the reported results of the potential immunogenicity of naïve allogeneic MSCs or their derived IPCs were conflicting. This discrepancy highlights the need to characterize the intricate mechanisms by which MSCs exert their immunomodulatory properties and identify the variables that may influence these functions.

**Figure 1:**
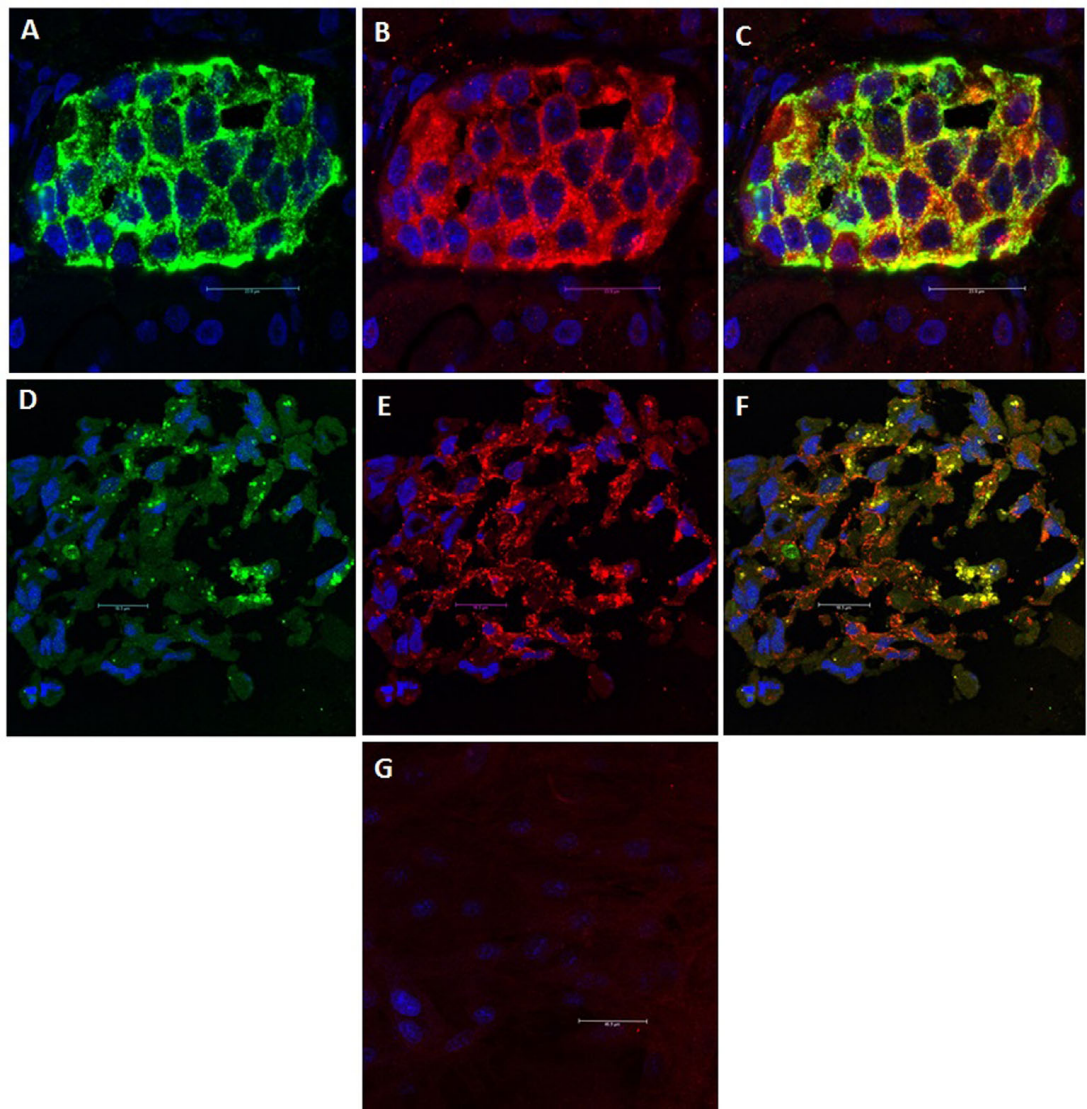
GAD65 Expression: GAD65 converts glutamic acid into GABA, which takes part in β-cell proliferation and regeneration and glucose homeostasis. **(A)** Human islets: Positive staining for insulin (Green). **(B)** Human islets: Positive staining for GAD65 (Red). **(C)** Human islets: Electronic merging revealed the presence of GAD65 within the majority of insulin-producing cells (Yellow). **(D)** MSC-derived IPCs: Positive staining for insulin (Green). **(E)** MSC-derived IPCs: Positive staining for GAD65 (Red). **(F)** MSC-derived IPCs: Electronic merging revealed the presence of GAD65 within the majority of insulin-producing cell (Yellow). **(G)** Negative staining for GAD65 in undifferentiated MSCs.

The current understanding is that islet cells are both the driver for and the target of the autoimmune T1DM. It is widely believed that genetic predisposition underlies individual susceptibility to the disease (34). However, monozygotic twins do not exhibit identical susceptibility to T1DM, which indicates the contribution of additional factors, such as metabolic stress (35), viral infection (34) and changes in the intestinal microbiota (36). As a result of impaired thymic education, islet-specific autoreactive lymphocytes migrate from the pancreas-draining lymph nodes to the islets, where they mediate β-cell destruction (35, 37). The detection of autoantibodies against islet proteins [insulin (IAA), glutamic acid decarboxylase 65 (GAD65), zinc transporter 8 (ZnT8) and insulinoma-associated antigen-2 (IA-2)] is believed to be the best predictor of the course and clinical manifestation of T1DM (38). However, the role of autoantibodies in disease pathogenesis is not yet well understood (39). In an immunohistological investigation in our laboratory, we observed that while undifferentiated MSCs did not express GAD65, differentiated MSCs did (**Figure 1**). GAD65 serves to convert glutamic acid into gamma aminobutyric acid (GABA) (38), which is thought to take part in β-cell proliferation and regeneration and glucose homeostasis (40). These findings suggest that MSC-derived IPCs exhibit some biological similarities to naïve islets and might be affected by the same events that result in T1DM. This conclusion raises a key question of whether the immunomodulatory functions of MSCs and their derived IPCs can overcome allogeneic responses and modulate the autoimmune pathways implicated in T1DM.

## MSCs and Immune System Components

Several reports have demonstrated that MSCs can modulate the activation and function of various immune cells belonging to both the innate and adaptive components of the immune system. It has also been shown that MSCs are capable of exerting their immunosuppressive effects especially in an inflammatory environment where they are activated by proinflammatory cytokines. Of these, IFN- γ is the most essential, acting synergistically with TNF-α. IL-1 and IL-17 can also boost MSC-mediated immunomodulation. It is worthy to note that different cytokines activate distinct signaling pathways and might differently regulate the mechanisms involved in the immunomodulatory properties of MSCs (41, 42).

### MSCs, T Lymphocytes and Their Effector Cells

*In vitro*, MSCs can suppress the proliferation of T-cells induced by allo-antigens or mitogens (43–46). This is primarily the result of cell division arrest at the G_0_/G_1_ phase of the cell cycle (47) and is thought to be mediated by cell-to-cell interactions along with the release of cytokines and soluble factors (48). MSCs are also capable of inducing apoptosis in activated T cells; a process associated with the conversion of tryptophan into kynurenine (49) or through the FasL-mediated pathway (50). In addition, MSCs exert suppressive actions on T cell differentiation towards the proinflammatory T-helper (Th) subsets Th1 and Th17 (51). In their experimental study, Luz-Crawford et al. reported that, in the presence of MSCs, significant reductions in the numbers of IFN-γ-producing CD4^+^ Th1 cells and IL-17-producing Th17 cells were observed (52). In addition, the researchers observed an increase in the proportion of FOXP3-expressing regulatory T cells (Tregs). Ghannam et al. reported that, in an inflammatory environment, MSCs triggered the regulatory properties of CD4^+^ Th17 cells. They maintained that this effect is achieved by downregulating the Th17 cell-specific transcription factor retinoic acid receptor-related orphan receptor gamma t (RORγt) and upregulating FOXP3 (53).

On another note, CD8^+^ cytotoxic T lymphocytes (CTLs) are activated upon interacting with peptides expressed on MHC class-I (54). Despite their expression of MHC class-I, MSCs do not activate CTLs (55). Instead, MSCs have been shown to suppress the differentiation of CTL precursors into effector CTLs. The early addition of MSCs to a mixed lymphocyte culture (MLC) significantly inhibited the lytic activity of CTLs against target cells. However, lysis was not affected when MSCs were added on or after day 3 (56), revealing the inability of MSCs to exert their inhibitory effects once the cytotoxic phase of CTLs had been reached.

### MSCs and Regulatory T Lymphocytes (Tregs)

Sakaguchi et al. isolated a unique population of CD4^+^CD25^+^ T cells that exhibited immunosuppressive properties. Later, this subpopulation was given the name Tregs (57). Based on their developmental origin, Tregs are classified into two major categories: those that develop naturally in the thymus (nTregs) and those that are induced peripherally during activation (iTregs). In contrast to nTregs, which express demethylated FOXP3, iTregs are inducible, and their FOXP3 is fully methylated (58). Several reports have demonstrated that MSCs are able to trigger Treg proliferation both *in vitro* and *in vivo* (59–61). Evidence has shown that this proliferation is induced and not the result of the expansion of nTregs (53, 62). To date, four mechanisms have been proposed for this process: cell-to-cell contact-dependent, soluble factor-dependent, antigen-presenting cell (APC)-dependent and exosome-dependent*. In vitro* studies by English and associates demonstrated that MSCs promoted Treg activation *via* a sequential process: cell-to-cell contact with CD4^+^ T cells which was followed by the release of PGE2 and transforming growth factor-beta (TGF-β) (63). Fallarino and colleagues claimed that the induction of a regulatory phenotype in naïve T cells was the result of tryptophan starvation and its catabolites (64). In addition, MSCs were shown to drive APCs towards a regulatory phenotype that promoted Treg activation through interleukin-10 (IL-10) secretion (65, 66). On the same note, Zhang et al. reported delayed rejection of allogeneic skin grafts in mice with polarization of CD4^+^ cells towards CD4^+^CD25^+^FOXP3^+^ Tregs as a function of MSC-derived exosomes (67). Infusion of those exosomes into a humanized mouse model of graft-versus-host reaction resulted in reduced mortality and increased numbers of Tregs (68). Although the potential of MSC-derived exosomes is promising, their role in Treg induction, in comparison to other mechanisms, has yet to be validated.

### MSCs and B Lymphocytes

In their *in vivo* experiments, Corcione et al. demonstrated that human BM-MSCs inhibited the proliferation of stimulated B cells and suppressed their differentiation to antibody-producing cells (plasmablasts) *in vivo* (69). The expression of the chemokine CXCR4 was preferentially downregulated, resulting in the retardation of B cell chemotaxis. Transwell experiments further confirmed that the inhibitory effect of MSCs on activated B lymphocytes was mediated by the release of soluble factors. In another study, Franquesa et al. reported that the activation of human adipose tissue-derived MSCs (hAT-MSCs) by inflammatory cytokines induced the expression of indoleamine-2,3-dioxygenase (IDO) and programmed death ligand 1 (PD-L_1_). The activated cells were then able to inhibit the proliferation of B cells *in vitro* (70). They also observed that hAT-MSCs had a direct effect, independent of T cells, on stimulated B cells, inhibiting their differentiation into plasmablasts and triggering the formation of regulatory B cells (Bregs). Luk and associates stressed the fact that the immunological conditions dictate the influence of MSCs on B cell functions (71). Under resting conditions, hAT-MSCs stimulated IL-10-producing Bregs. Whereas, in an inflammatory microenvironment, they inhibited B cell proliferation.

### MSCs and Natural Killer (NK) Cells

Several studies have proven that MSCs suppress NK cell proliferation and cytokine production (72). In such cases, the ratio of MSCs to NK cells is critical; the suppressive action requires a high MSC-to-NK cell ratio (73). It was also confirmed that MSCs induce CD73 upregulation in NK cells. As a result, adenosine monophosphate (AMP) is converted into adenosine, which is an anti-inflammatory molecule (74). Conversely, at low ratios, MSCs were shown to support NK cell proliferation (75). In a more recent study by Hu and associates, NK cells were cocultured with MSCs at a ratio of 10:1 (76). The authors noted that MSCs did not alter NK proliferation but reduced their IFN-γ production and lytic functions. They maintained that these effects were mediated by IDO and PGE2.

### MSCs and Dendritic Cells (DCs)

Monocytes and CD34^+^ hematopoietic progenitor cells (HPCs) can differentiate into macrophages or DCs, the most potent APCs that can activate T cells and initiate immune responses. It was reported that MSCs prevent the differentiation into mature DCs from either source (77). Transwell experiments by Nauta et al. indicated that the underlying mechanisms involved the release of soluble factors, mainly IL-6 and IL-10 (78). Similarly, the coculture of mature DCs with MSCs resulted in reduced expression of CD80, CD83 and CD86, along with the downregulation of IL-12, indicating that DCs were driven into an immature state (45). Additionally, Li et al. showed that direct coculture of MSCs with adult CD34^+^ HPCs induced the generation of alloantigen-specific Tregs (79). The authors suggest that this action involved direct contact between MSCs and HPCs and was mediated by activation of the Notch signalling pathway. These MSC-primed DCs can, therefore, be considered regulatory since they express high levels of IL-10, inhibit the proliferation of alloreactive T cells and induce the generation of allogen-specific Tregs.

### MSCs and Macrophages

Macrophages have both proinflammatory and anti-inflammatory roles and can thus take part in tissue inflammation and damage on one hand, and repair and healing on the other. Several *in vitro* studies have demonstrated that coculturing macrophages with MSCs resulted in macrophage polarization towards an immunomodulatory M2 phenotype, that secreted high levels of IL-10 (80–82). To exert their therapeutic functions, MSCs can be primed with proinflammatory TNF-α and anti-inflammatory IL-10 cytokines produced by M1 and M2 macrophages, respectively (83). Evidence was provided that induced MSCs secrete regulatory molecules such as PGE2, which promote M2 macrophages and suppress M1 macrophages. Li et al. showed that the interaction between MSCs and M1 macrophages is not only paracrine but also contact-dependent *via* the interaction between CD200 expressed by MSCs and its receptor CD200R on the M1 macrophage surface (84). In the context of the subject matter of this review, it is of interest that macrophages are the main immune cell component of murine and human islets (85). Dalmas and associates reported that islet-associated macrophages are in close proximity to β-cells and regulate insulin secretion through the production of retinoic acid (86). According to Weitz et al., macrophages reside in perivascular regions and produce anti-inflammatory IL-10, which is notably reduced in obese/diabetic individuals (87). Collectively, these findings can provide further insight into MSC-based therapy for T1DM.

### MSCs and Neutrophils

A number of studies have confirmed the preservative role of MSCs on neutrophil viability and function (88–90). Such protective effect is mediated by MSC release of IL-6. In contrast to their suppressive action on other immune cells, MSC-mediated protection of neutrophils was proven to be vital for maintaining neutrophil phagocytic and bactericidal functions.

## Mechanisms of MSC-Mediated Immunomodulation

**Figure 2:**
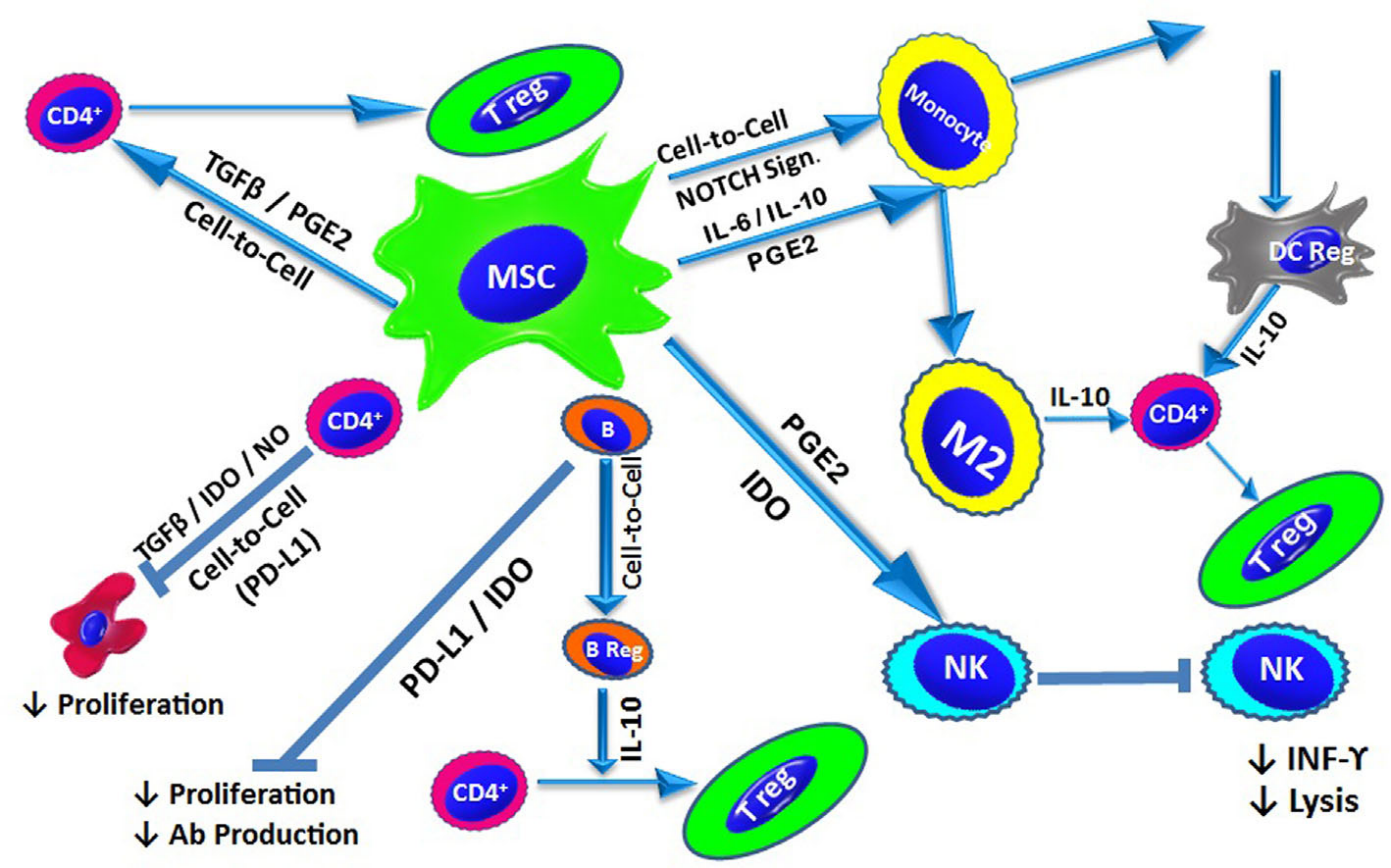
MSC-Mediated Immunomodulation: Under inflammatory conditions, MSCs exert their immunomodulatory effect *via* cell-to-cell contact and/or the release of soluble factors. In addition to contact-dependent inhibition, MSCs suppress T cell proliferation by secreting IDO, NO and TGF-β. MSCs also modulate naïve T cells to generate Tregs through cell-to-cell contact, as well as *via* TGF-β and PGE2. B cell proliferation and antibody production is inhibited by IDO and PD-L_1_. Through direct interactions, MSCs also promote the generation of IL-10-producing Bregs. MSCs affect the innate immune system and suppress NK cell proliferation and cytokine secretion, a process mediated by IDO and PGE2. MSCs also inhibit the differentiation of monocytes into DCs *via* the release of IL-6 and IL-10. Additionally, MSCs can drive DCs towards an IL-10-producing tolerogenic phenotype through the activation of Notch signalling. MSCs also direct macrophages towards an anti-inflammatory phenotype (M2) *via* the action of IL-10. IL-10 production from *via* different pathways triggers the generation of Tregs.

The mechanisms underlying the immunomodulatory properties of MSCs are interdependent and involve soluble factor secretion and cell-to-cell contact (**Figure 2**).

### Soluble Factors

#### Transforming Growth Factor-β (TGF-β)

As early as 1999, TGF-β was recognized as a cytokine that was secreted by MSCs and plays a role in the regulation of the immune response (91). Tomic et al. reported that MSCs isolated from dental pulp produced TGF-β, which suppressed the proliferation of peripheral blood mononuclear cells (PBMCs). This action was blocked by the addition of anti-TGF-β antibodies (92). In a study by Nasef et al., the suppressive effect of MSC-produced TGF-β on T cell proliferation was shown to be largely dependent on cell-to-cell contact (93). It was also noted that TGF-β could modulate the differentiation of T-helper subsets by promoting Th2 and inhibiting Th1 and Th17 responses (94). Moreover, English and colleagues demonstrated that BM-MSCs induced FOXP3^+^ Tregs *via* TGF-β *in vitro* (63). This finding was confirmed by Wang et al., who demonstrated that TGF-β-inactivated hUC-MSCs lacked the ability to generate Tregs *in vitro* (95). Finally, TGF-β was also reported to promote anti-inflammatory M2 macrophages (96).

#### Indoleamine-2, 3-Dioxygenase (IDO)

Using Western blot analysis, Meisel et al. demonstrated that hMSCs do not constitutively express IDO. Instead, IDO protein expression by MSCs is induced by IFN-γ in a dose-dependent manner (97). Mounayar and colleagues have identified phosphoinositide 3-kinase alpha (PI3Kα) as a major regulatory of IFN-γ-induced IDO expression by MSCs. This effect was shown to be mediated *via* the signal transducer and activator of transcription 1 (STAT1) (98). Of interest, Li et al. observed that while low concentrations of cytokines were sufficient to upregulate chemokine secretion, they were not enough to induce significant expression of IDO in mammals and nitric oxide (NO) in rodents (99). Chemokine-recruited lymphocytes accumulate in the vicinity of MSCs without being suppressed. According to Wang et al., MSCs can be immunosuppressive in strong inflammatory conditions. Paradoxically, with weak inflammation, their immunomodulatory function is abrogated and immune responses are enhanced (100). Accordingly, the authors described IDO levels as an “on-off switch” dictating the immunomodulatory outcome of MSCs. IFN-γ-mediated IDO expression by hAT-MSCs resulted in the inhibition of lymphocyte proliferation (101). IDO-mediated tryptophan depletion, *via* its catabolic conversion to kynurenine, was shown to be responsible for this inhibition (102). Abrogation of NK cell proliferation and maturation was also confirmed to be the result of MSC-derived IDO (103). In an experimental study by Ge et al., MSC infusion 24 hours post-allogeneic kidney transplantation in mice enhanced graft tolerance. The tolerant recipients exhibited an increase in kynurenine levels as well as high frequencies of tolerogenic DCs and Tregs. On the other hand, renal allograft recipients treated with MSC infusion concomitant with the IDO inhibitor 1-methyltryptophan did not attain graft tolerance. These findings confirm that functional IDO was necessary to induce the observed immunomodulation (104).

#### Nitric Oxide (NO)

Inducible NO synthase (iNOS) has been detected in MSCs. In the presence of activated T cells, there is notable dose-dependent production of NO by MSCs. NO-mediated suppression of T cell proliferation is the result of the inhibition of STAT-5 phosphorylation, which is required for cell proliferation. In Transwell experiments, this suppression was reduced, suggesting that this function depends on cell-to-cell contact (105). It is worth noting that MSC-mediated immunosuppression is not similar among different species. While this effect is mediated by NO in mice, it is mediated by IDO in humans (106).

#### Prostaglandin E2 (PGE2)

Baratelli and colleagues demonstrated that PGE2 induced expression of the Treg-specific transcription factor FOXP3 in CD4^+^CD25^+^ Tregs (107). English et al. reported that the action of PGE2 was preceded by cell-to-cell contact (63). The resulting Tregs acted similarly to naturally developed nTregs. PGE2 was also shown to be involved in the suppression of monocytic differentiation into DCs (108), as well as the inhibition of proliferation and cytotoxic activity of NK cells (109).

#### Interleukin 10 (IL-10)

IL-10 is another main immunosuppressive cytokine associated with MSCs, and its expression can be further enhanced by PGE2 (110). IL-10 was demonstrated to inhibit APC maturation, suppress Th17 generation and promote Treg proliferation (111).

#### Programmed Death Ligands 1 and 2 (PD-L_1_ and PD-L_2_)

MSCs constitutively express the programmed death ligands PD-L_1_ and PD-L_2_, which are upregulated in the presence of inflammatory cytokines. Through cognate contact between PD-L_1_- and PD-L_2_-expressing MSCs and PD-1-expressing lymphocytes, the kinase activity of the T cell receptor is disrupted (112). This disruption inhibits T cell proliferation and induces T cell anergy and apoptosis (113, 114). Davies et al. provided evidence that in addition to the surface expression of PD-L_1_ and PD-L_2_ by MSCs, these factors are also secreted into the microenvironment upon stimulation with IFN-γ and TNF-α. Based on these findings, it was suggested that stimulated MSCs can exert their immunosuppressive function by cell contact, as well as by contact-independent pathways (115). Fiorina and associates shed light on the important role of PD-L_1_ in immunomodulation. They reported that, compared to NOD MSCs, BALB/c or NOR (NOD-related, diabetic resistant mice) MSCs express higher levels of PD-L_1_. Administration of BALB/c MSCs achieved temporary reversal of hyperglycemia in 90% of NOD mice. However, in this setting, soft tissue and visceral tumor were observed in the recipient NOD mice (116). In a further investigation by Jurewicz et al., NOR MSCs were able to suppress NOD CD4^+^ T cell proliferation *via* PD-L_1_, and inhibit the generation of proinflammatory DCs through an IL-6-dependent mechanism. NOR MSC-based treatment of experimental type 1 diabetic NOD mice resulted in reversal of hyperglycemia. It was concluded that congenic MSC therapy can be useful in future clinical trials for treatment of type 1 diabetes (117). Using human placenta-derived MSCs (hP-MSCs), Gu and associates observed that these cells expressed not only PD-L but also Fas ligands (FasL) (118). Neutralizing antibodies against PD-L_1_ and FasL significantly interfered with the suppressive effect of hP-MSCs on T cell proliferation. However, anti-FasL but not anti-PD-L_1_ antibodies suppressed activated T cell apoptosis. The authors concluded that both ligands played significant but different roles in hP-MSC-mediated immunomodulation.

### Cell-to-Cell Contact: Adhesion Molecules and Chemokines

Interactions between cell surface receptors and their ligands are central for cell attachment, communication and function. Under inflammatory conditions, in addition to secreting anti-inflammatory cytokines and soluble factors, MSCs express adhesion molecules and chemokines that enable them to establish cell-to-cell contact with lymphocytes (119). Majumdar et al. identified distinct cell surface molecules expressed on MSCs, including intracellular adhesion molecule (ICAM)-1 and ICAM-2 and vascular cell adhesion molecule (VCAM)-2 (120). Stimulation of MSCs with IFN-γ and TNF-α upregulated their ICAM-1 surface expression, increasing their affinity towards activated T cells. Working with murine BM-MSCs, Ren and associates observed that stimulating MSCs with IFN-γ with TNF-α or IL-1 upregulated ICAM-1, as well as VCAM-1, increasing their affinity towards activated T cells. Genetic deletion or functional blockade of these adhesion molecules abrogated the immunosuppressive effect of MSCs *in vitro* and *in vivo* (121). In addition, stimulation of MSCs was shown to induce robust expression of several chemokines (122), of which the majority were CXC chemokine receptor 3 (CXCR3) and CC chemokine receptor 5 (CCR5) ligands. Using time-lapse microscopy, it was proven that activated T cells accumulate in close proximity to cytokine-activated MSCs. Pharmacological or genetic blockade of CXCR3 or CCR5 prevented cell migration and inhibited MSC-induced immunosuppression.

Recent studies have shed more light on mechanisms involved in contact-dependent signalling between hAT-MSCs and activated T cells. Matula et al. provided evidence that the exchange of cytoplasmic material between these cells was mediated by tunnelling nanotubes and extracellular vesicles exclusively derived from T cells (123). As a result, the properties of MSCs shift towards an anti-inflammatory phenotype associated with the production of soluble immunosuppressive molecules, including PGE2.

In summary, soluble factors together with cell-to-cell contact create a microenvironment in which the immunomodulatory functions of MSCs are rendered more pronounced.

## Factors Influencing the Immunomodulatory Functions of MSCs

Numerous factors can alter the immune properties of MSCs, which may explain, at least in part, the contrasting results reported in the literature. In addition, it would be valuable to identify these variables to circumvent those that hinder and exploit others that promote the immunomodulatory properties of MSCs if an application of this function is contemplated.

MSCs derived from different sources exhibit distinct expansion, differentiation, paracrine and immune characteristics. In a comparative study, Ribeiro and colleagues evaluated the immunosuppressive functions of AT-MSCs, BM-MSCs and umbilical cord matrix-derived MSCs (UCM-MSCs). AT-MSCs were shown to pose a stronger inhibitory effect on T cell proliferation and blocked T cell activation at an earlier stage than BM-MSCs or UCM-MSCs (124). Additionally, several publications have reported that AT-MSCs are more potent suppressors of DC differentiation and exhibit a higher immunomodulatory capacity than BM-MSCs (125, 126). Valencia et al. compared the immunomodulatory properties of AT-MSCs and BM-MSCs from the same donor to overcome any heterogeneity resulting from donor-to-donor variations. The researchers concluded that AT-MSCs and BM-MSCs have comparable immunomodulatory features. Selection of either cell type would, therefore, depend on other factors, including availability and MSC density in the tissue samples (127).

Donor age also has an impact on the therapeutic potential of derived MSCs. The yield of hMSCs obtained by marrow aspiration is likely to decline with age. MSCs derived from younger individuals were proven to have higher proliferation rates with lower levels of oxidative stress-mediated damage (128). Wagner and associates maintained that the changes upon ageing were associated with the replicative senescence of MSCs (129).

In a study by Serena et al., the immunomodulatory capacities of AT-MSCs derived from lean, obese and diabetic donors were compared. They reported that obese-derived AT-MSCs or those from type 2 diabetic patients were less effective in suppressing lymphocytic proliferation, inducing M2 macrophages and releasing TGF-β than those from healthy controls (130). Metabolic changes resulting from the diabetic microenvironment can provide an explanation (131). This conclusion suggests the question of whether such changes can affect the therapeutic function and immunomodulatory properties of MSC-derived IPCs after transplantation in diabetic patients. In an experimental study by our group, chemically-induced diabetes in nude mice did not interfere with the *in vivo* maturation of transplanted hBM-MSC-derived IPCs (132).

The methods utilized for MSC isolation, expansion and preservation can also significantly affect the quality and potential therapeutic efficacy of MSCs. Yoo et al. compared the conventional gradient centrifugation technique with a sub-fractionation culture method and showed that cloned hMSCs were more efficient than conventionally isolated MSCs in regulating graft-versus-host disease (133). Optimization of MSC culture conditions also has a critical impact on MSC functionality. MSCs cultured under hypoxic conditions are forced to switch to anaerobic metabolism. The resulting lactate directs macrophages towards an anti-inflammatory phenotype (134). It was also noted that MSCs in 3D culture exhibit stronger immunosuppressive properties, and the secretion of immunomodulatory molecules was enhanced (135). Evidence has shown that MSCs expanded in media containing fetal bovine serum can develop anti-fetal calf serum antibodies upon *in vivo* application (136). It is also worth noting that MSCs expanded in platelet lysate showed decreased inhibitory capacity on T and NK cell proliferation (137). In a study by Liu et al., the relationship between MSC passage number and their immunomodulatory function was investigated (138). The authors demonstrated that with increasing passage number, there was a gradual loss of MSC immunomodulatory properties. These changes became statistically significant after passage 10. Cryopreservation, followed by thawing before utilization, could also result in low cell viability or induce heat shock responses that may account for the reduced modulatory functionality of MSCs (139).

The chosen route of administration may also influence the immune function of MSCs. The immunogenicity of allogeneic MSCs transplanted by different routes in diabetic mice was compared by Gu et al. The researchers observed that cells transplanted under the pancreatic capsule evoked an immune reaction. Conversely, MSCs infused through the tail vein retained their immunosuppressive capabilities (140).

## Enhancement of the Immunomodulatory Functions of MSCs

There have been increasing attempts to enhance the immunomodulatory functions of MSCs and achieve improved therapeutic outcomes (141, 142). These measures include *in vitro* pretreatment, coadministration of mTOR inhibitors, Treg utilization and gene manipulation.

Since the immunosuppressive properties of MSCs are triggered in an inflammatory environment, preconditioning with proinflammatory cytokines has been used to enhance these functions. Increased secretion of PGE2 and TGF-β and induced expression of PD-L1 were observed in IFN-γ-primed MSCs (143, 144). On this basis, IFN-γ-conditioned MSCs were experimentally used to treat some immune-associated disorders (145, 146). Oliveira and associates evaluated the survival of untreated allogeneic MSCs compared to those pretreated with IFN-γ and TNF-α following transplantation under the renal capsule of immunocompetent mice (147). *In vivo*, both groups of cells provoked an immune response with inflammatory cell infiltration at the transplantation site and consequent graft loss. Combined transplantation of preactivated cells and allogeneic islets was associated with marginal prolongation of graft survival. Yoshihara and associates differentiated human iPSCs into what they termed human islet-like organoids (HILOs). Immune-evasive HILOs were initially generated by transfection with a PD-L_1_-expressing lentivirus, and later by short repeated exposures to IFN-γ to induce sustained expression of PD-L_1_. Transplantation of these HILOs in immune-competent streptozotocin (STZ)-induced diabetic mice reduced blood glucose levels for more than 40 days, while the functional efficacy of untreated HILOs was progressively lost. Similar results were seen upon transplantation in diabetic humanized mice. Surgical removal of the cell-bearing kidneys was followed by an abrupt loss of glycaemic control. Examination of the explanted specimens revealed an increase in the number of insulin-expressing cells and a decrease in lymphocytic infiltration. The authors maintained that this approach could provide a promising alternative to cadaveric-dependent sources for the treatment of diabetes (148). On the other hand, Sivanathan and associates pointed out that the upregulated expression of MHC in allogeneic IFN-γ-stimulated MSCs may boost their immunogenicity and dampen their immunomodulatory functions (149). Coadministration of immunosuppressive drugs, rapamycin in particular, may offset this phenomenon *in vivo*.

Battalgia and colleagues reported that rapamycin, an mTOR inhibitor, selectively expanded murine nTregs *in vitro*. The expanded nTregs suppressed the proliferation of syngeneic T cells *in vitro* and prevented allograft rejection *in vivo* (150). Wang and colleagues provided evidence that rapamycin enhanced the immunomodulatory functions of MSCs by upregulating COX-2 expression and inducing PGE2 release. The authors noted that prolonged exposure to rapamycin did not support this effect and suggested that only short-term incubation of MSCs with rapamycin could improve immunomodulation and represent a novel therapeutic tool (151).

The use of Tregs can provide an additional tool for enhanced immunomodulation. Tregs are usually isolated from peripheral blood and expanded ex vivo (152). By providing a tool to generate location-specific or antigen-specific Tregs, chimeric antigen receptor (CAR) gene engineering can potentially boost the specific suppressive capacity of Tregs, circumventing off-target immunosuppression (153).

Gene manipulation involves transduction of the appropriate gene or genetic engineering. Garcia-Ocana and associates transduced the hepatocyte growth factor gene *via* an adenoviral vector into murine islets *in vitro*. Upon transplantation of these cells in STZ-induced diabetic mice, improvements in graft survival and blood glucose control were observed (154). Qi et al. used a lentiviral vector to overexpress the FOXP3 gene in rat MSCs. Following liver transplantation, FOXP3^+^ MSCs from the same strain or a third-party rat were intravenously infused. The authors reported that FOXP3^+^ MSCs induced liver allograft tolerance (155). Again, Niu and colleagues prepared syngeneic rat MSCs by transduction with IL-10. These cells were intravenously injected into a rat model after liver transplantation. The results suggested that IL-10-engineered cells had the potential to overcome rejection of the transplanted liver (156). Collectively, these results indicate that the engraftment of transduced MSCs can promote immunomodulation and support cell or organ transplantation. However, these procedures rely on the use of viral vectors, which impose the limitations of possible oncogene transactivation and a lack of physiological expression to allow monitoring.

Recently, gene editing has emerged as a powerful alternative approach (157). Moghadan and associates developed a CRISPR-based system that could effectively modulate the immune responses of the host (158). To this end, the authors made use of the transcriptional repressors heterochromatin protein 1 (HPIα) and Kruppel-associated box protein (KRAB). With a gRNA aptamer, these repressors were fused to a nuclease-competent CRISPR containing a truncated gRNA. This complex could repress the myeloid differentiation protein (Myd88) gene, which encodes the Myd88 protein that is involved in innate signalling. This system was shown to effectively downregulate IgG production against an adeno-associated virus in mice. The authors concluded that CRISPR-mediated suppression of endogenous Myd88 could modulate host immune responses and be used as a therapeutic modality for immune modulation.

The inhibitory CD47 ligand is an efficient immunomodulator and has been proven to be upregulated in cancer (159, 160). In a series of recent experiments, Deuse et al. noted that CD47 overexpression prevented NK cells from killing MHC class I- and II-deficient targets in mice (161). The researchers then provided evidence that the immune-inhibitory function of CD47 is exerted by ligation to signal regulatory protein α (SIRPα) on NK cells. They also observed that *Sirpα* expression in murine NK cells and its CD47 binding were strongly upregulated by IL-2 in a dose-dependent manner. Similar observations were reported in human NK cells. Since blocking either CD47 or SIRPα rendered human HLA-deficient endothelial cells susceptible to primary NK cell cytotoxicity, it was concluded that in humans, SIRPα was essential for the transmission of the CD47-induced inhibitory signal to NK cells. The authors estimated the necessary threshold for CD4 expression that could inhibit innate immune responses to be ≈3.5-fold higher than basal values. Furthermore, cloned rhesus monkey CD47 (rhCD47) was transduced by a lentivirus into human HLA-deficient endothelial cells. *In vitro*, human cells were spared the deleterious effects of rhesus NK cells and macrophages. This finding demonstrated that rhCD47 expression could inhibit rhesus monkey-derived innate cells in a xenogeneic setting. Collectively, these experiments suggest that the generation of non-immunogenic stem cells is possible. Such cells can evade immune responses and differentiate into functional somatic cells following transplantation. Genetically modified cells, however, carry potential risks. It is important to demonstrate that in addition to the expected benefits, these cells do not give rise to tumorigenic or otherwise harmful cells (32).

Notably, MSC-derived exosomes transfer biomolecules to target tissues or cells. The intrinsic contents of exosomes are determined by the biological components of the parent cell cytoplasm (162). Accordingly, preconditioning or genetic engineering of MSCs can improve the therapeutic efficacy of their derived exosomes. Coadministration of these exosomes combined with allogeneic MSC-derived IPCs could provide an additional tool for enhancing their immunomodulatory properties.

In a different approach, Al-Daccak and Charron suggested that banking allogeneic stem cells could provide an opportunity to select HLA-compatible donors and avoid detrimental mismatches (163). The size of such a donor bank would depend on the frequencies of HLA haplotypes. Within the same ethnic background, it is estimated that storing ≈100 samples would allow for the selection of suitable donors. The authors also proposed identifying, those with an immunomodulatory benefit among the permissive matches. Guan et al. suggested that the potency of the immunomodulatory capacity of MSCs can be evaluated (164). Following pretreatment with IFN-γ, the expression of intracellular IDO and surface expression of PD-L_1_ can be determined. Choosing donors with the highest expression levels is an additional optimization tool (165). Moreover, a Luminex-based solid-phase assay could be used to detect and minimize the risk of donor-specific antibodies (166). Collectively, these measures guide the identification of immune-educated, low-risk, high-benefit donors.

## Concluding Remarks

- Pluripotent stem cell-derived IPCs are immunogenic. Their transplantation requires encapsulation within an immunoisolation device and has inherent limitations.
- It is possible, though unlikely, to obtain autologous MSCs from type 1 diabetic patients if the patient’s own umbilical cord-derived MSCs have been previously stored.
- In the clinical setting, the use of allogeneic cells is more practical. Human AT-MSCs are widely available from cosmetic surgeries and should not be wasted. These cells can be stored, and their HLA type can be identified for selecting a compatible donor.
- To date, studies of the immunogenicity of allogeneic hMSC-derived IPCs have been rare, confusing and non-informative. Transplantation into a humanized mouse model can provide important clues.
- Based on experiences with allogeneic cardiac progenitors, the selection of low-risk and high-benefit donor cells should be explored when cell therapy for T1DM is contemplated.
- The pre-existing destructive autoimmune environment in T1DM should not be overlooked. In this context, the immunomodulatory properties of allogeneic hMSC-derived IPCs can be enhanced by pretreatment with inflammatory cytokines. In addition, the use of adjuvant immunosuppression may be necessary. Sirolimus, an mTOR inhibitor, would be the agent of choice. In addition to its well-known immunosuppressive effect, sirolimus can also enhance immunomodulation through the induction of COX-2 and PGE2 expression.
- Gene manipulation promises the generation of stem cells that can evade immune recognition, which, provided that they do not induce untoward effects, presents an alternative enhancement strategy.
- In any case, the ultimate cell therapy for type 1 diabetic patients should be as good as or better than the ever improving closed-loop insulin delivery systems.

## Author Contributions

AR: Revised the manuscript and arranged the references.BE: Edited the manuscript and verified the references. MK: Revised the manuscript and added to the section on macrophages and cell-to-cell contact. SK: Carried out the immunohistological study. AI: Revised the section on immunomodulation. RM: Revised the manuscript and edited the section on the special case of stem cell-derived IPCs. MG: Wrote the original draft and revised the final manuscript. All authors contributed to the article and approved the submitted version.

## Conflict of Interest

The authors declare that the research was conducted in the absence of any commercial or financial relationships that could be construed as a potential conflict of interest. 
